# The Uremic Toxin Indoxyl Sulphate Enhances Macrophage Response to LPS

**DOI:** 10.1371/journal.pone.0076778

**Published:** 2013-09-30

**Authors:** Simona Adesso, Ada Popolo, Giuseppe Bianco, Rosalinda Sorrentino, Aldo Pinto, Giuseppina Autore, Stefania Marzocco

**Affiliations:** Department of Pharmacy, School of Pharmacy, University of Salerno, Fisciano (SA), Italy; Institut national de la santé et de la recherche médicale (INSERM), France

## Abstract

Indoxyl sulphate (IS) is a protein-bound uremic toxin that results from the metabolism of dietary tryptophan normally excreted by kidney through the proximal tubules. Thus the toxin accumulates in the blood of patients with impaired renal function such as in chronic kidney disease (CKD). High IS serum levels in patients with CKD suggest its involvement in CKD progression and in the onset of complications. Its presence in plasma is also a powerful predictor of overall and cardiovascular morbidity/mortality. IS is a well known nephrovascular toxin but very little is known regarding its effects on the immune system and in particular during inflammation. In this study we examined the effect of IS on macrophage activation in response to lipopolysaccharide from *E. coli* (LPS), a gram negative bacterial endotoxin associated with inflammation and septic shock. To simulate the uremic condition, J774A.1 macrophages were incubated with IS at concentrations observed in uremic patients (1000–62.5 µM) both alone and during LPS challenge. IS alone induced release of reactive oxygen species (ROS), through a mechanism involving pro- and anti-oxidant systems, and alteration in intracellular calcium homeostasis. When added to J774A.1 macrophages in presence of LPS, IS significantly increased the nitric oxide (NO) release, inducible nitric oxide synthase (iNOS) and cycloxygenase-2 (COX-2) expression. IS pre-treatment was also associated with an increase in tumor necrosis factor-α (TNF-α) and interleukin-6 (IL-6) production by macrophages stimulated with LPS. Mechanistic studies revealed that IS increased LPS-induced NF-kB nuclear translocation, ROS release and altered calcium concentrations, mainly because of mitochondrial calcium overloading. Moreover also in primary mouse peritoneal macrophages IS enhances the inflammatory response to LPS increasing ROS, NO, iNOS, COX-2, TNF-α, IL-6 and NF-kB levels.

This study provides evidences that IS stimulates macrophage function and enhances inflammatory reasponse associated with LPS, thus contributing to altered immune response dysfunctions observed in CKD.

## Introduction

Chronic kidney disease (CKD) progression leads to the dysfunction of multiple organs with clinical features constituting the uremic syndrome that, despite pharmacological and dialytic treatment remains associated with multiple complications [1]–[3]. This syndrome is attributed to the progressive retention of a large number of compounds which, under normal conditions, are excreted by healthy kidneys. These compounds are called uremic toxins because of their harmful effects in various physiological functions in CKD patients [1], [2].

The uremic toxin indoxyl sulphate (IS) is a protein-bound uremic toxin that results from the metabolism of dietary tryptophan. Tryptophan is metabolized into indole by intestinal bacteria and after intestinal absorption, is further converted into IS in the liver [4]. In healthy subjects the toxin is excreted by the kidney proximal tubules, whereas in patients with impaired renal function IS accumulates in the blood. High serum IS levels in patients with CKD could be responsible for CKD progression and complications related to this pathology [5], [6]. The development of CKD is associated with a significant increase in all-cause mortality [7], [8]. The main factors responsible for the increased risk of morbidity and mortality in patients with CKD are cardiovascular dysfunctions and infections, both linked to changes in immune responses [9], [10]. In uremia, a diminished immune defence contributes to the high prevalence of infections, whereas pre-activation and priming of immune cells leads to inflammation and consequently to cardiovascular disease.

There are multiple causes for the immune reaction that characterizes dysfunction related to uremia and dialysis. They may include accumulation of uremic toxins, strongly present in CKD and only partially corrected by dialysis, dialysis-related factors such as interactions between blood and dialyzer, endotoxin presence in water, access-related infections, and peritoneal dialysis solutions with high glucose concentration, low pH, and the presence of glucose degradation products represent chronic stimuli to the inflammatory response [11]. Oxidative stress and inflammation are crucial for defence against infections, but they initiate a number of deleterious effects, such as intracellular calcium impairment and cytokine production, if not properly regulated [12]. Oxidative stress increases in parallel with the progression of CKD and correlates with the level of renal function [13]. Furthermore, the antioxidant systems are severely impaired in CKD patients and worsen progressively with the degree of renal failure [14]. Biomarkers for oxidative stress, such as advanced oxidation protein products and myeloperoxidase activity, and the ones for inflammation, such as high sensitivity C reactive protein and cytokines, are interrelated in CKD [15]. Thus the pharmacological manipulation of inflammation and the control of oxidative stress are of particular importance in the uremic syndrome. Uremic toxins are responsible for many uremia-associated dysfunctions and among them there is an altered immune response [16]–[20]. IS is well known as a CKD-associated nephro-vascular toxin but very little is known regarding its effect on immune response [21]. In particular, to our knowledge, no study has been conducted so far on the effect/s of IS on macrophages, representing a relevant component of cell mediate defence in LPS-induced inflammatory conditions, a condition which often occurs in CKD patients.

In this study we report the effect of IS on macrophage function both under control conditions and in the presence of LPS, an activator of pro-inflammatory patterns. These data further correlate the exacerbated morbidity of patients with renal dysfunctions to macrophage-mediated pro-inflammatory patterns.

## Materials and Methods

### Reagents

Unless stated otherwise, all reagents and compounds were purchased from Sigma Chemicals Company (Sigma, Milan, Italy).

### Cell culture

J774A.1 murine monocyte macrophage cell line (American Type Culture Collection, Rockville, MD), was grown adherent to Petri dishes with Dulbecco's modified Eagle's medium (DMEM) supplemented with 10% foetal calf serum (FCS), 25 mM HEPES, 2 mM glutamine, 100 u/mL penicillin and 100 mg/mL streptomycin at 37°C in a 5% CO_2_ atmosphere. For our experiments, we referred to the list of uremic toxins provided by the European Uremic Toxin Work group and thus worked considering an IS range concentrations found in CKD serum patients [22].

### Primary murine peritoneal macrophages

Female C57BL/6 mice (6–8 weeks; Harlan Laboratories, Udine, Italy) were fed a standard chow diet and housed under specific pathogen-free conditions at the University of Salerno, Department of Pharmacy. All animal experiments were performed under protocols that followed the Italian and European Community Council for Animal Care (DL.no 116/92). Peritoneal cells were harvested by means of lavage of the peritoneum with 5 ml of EDTA 0.5 mM plated and allowed to adhere for 2 h at 37°C in a 5% CO_2_ atmosphere. Subsequently, non-adherent cells were removed and RPMI 1640 medium with 10% FBS was added. The cells were maintained in culture for 24 h at 37°C in a 5% CO2 atmosphere before experiments.

### Antiproliferative activity

Cells (5×10^4^/well) were plated on 96-well plates and allowed to adhere for 4 h. Thereafter, the medium was replaced with fresh medium alone or containing serial dilutions of IS (1000–62.5 µM), and incubation was performed for 24 h. Cell viability was assessed using the MTT assay as previously reported [23], [24]. Briefly, 25 µL of MTT (5 mg/mL) were added and cells were incubated for an additional 3 h. Thereafter, cells were lysed and the dark blue crystals solubilised with 100 µL of a solution containing 50% (v∶v) N,Ndimethylformamide, 20% (w∶v) SDS with an adjusted pH of 4.5. The optical density (OD) of each well was measured with a microplate spectrophotometer (Titertek Multiskan MCC/340-DASIT) equipped with a 620 nm filter. Macrophage viability in response to treatment with IS, was calculated as: % dead cells =  100×[(OD treated/OD control) ×100].

### Measurement of intracellular reactive oxygen species (ROS) and mithocondrial superoxide evaluation with MitoSOX Red

ROS formation was evaluated by means of the probe 2′,7′-dichlorofluorescin-diacetate (H_2_DCF-DA) by modifying a previously reported method [25]. H_2_DCF-DA is a non-fluorescent permeant molecule that passively diffuses into cells, where the acetates are cleaved by intracellular esterases to form H_2_DCF and thereby traps it within the cell. In the presence of intracellular ROS, H_2_DCF is rapidly oxidized to the highly fluorescent 2′,7′-dichlorofluorescein (DCF). Briefly, J774A.1 and mouse peritoneal macrophages cells were plated at a density of 3.0×10^4^ cells/well into 24-well plates. Cells were allowed to grow for 24 h; thereafter, the medium was replaced with fresh medium and cells were stimulated with IS (1000-62.5 µM) at the indicated times in figures. In some experiments either diphenyleneiodonium chlorhyde (DPI; 10 µM) or N-acetylcysteine (NAC; 20 mM) was added 1 h before IS. In other setting of experiments IS was added 1 h before and simultaneously to LPS (1 µg/ml) before ROS detection at the indicated time in figures. Cells were then collected, washed twice with phosphate buffer saline (PBS) buffer and then incubated in PBS containing H_2_DCF-DA (10 µM) at 37°C. After 45 minutes, cells fluorescence was evaluated using a fluorescence-activated cell sorting (FACSscan; Becton Dickinson) and elaborated with Cell Quest software.

In order to detect superoxide release from mitochondria in some experiments after cell treatment, as previously described, MitoSOX Red was used. MitoSOX Red (2.5 µM), was added for 10 min before fluorescence evaluation by means of flow cytometry. This indicator is a fluorogenic dye for highly selective detection of superoxide in the mitochondria of live cells and, once targeted to the mitochondria it is oxidized by superoxide and exhibits red fluorescence. MitoSOX is readly oxidized by superoxide but not by other ROS-generating systems.

Nitrite determination and Western blot analysis for inducible nitric oxide synthase (iNOS),, cicloxygenase-2 (COX-2) and IkB-α expression.

Macrophages were seeded in P60 plates (1.8×10^6^ for J774A.1 and 4×10^6^ for peritoneal macrophages/well) and allowed to adhere. Thereafter, the medium was replaced with fresh medium and cells were treated with IS (1000–62.5 µM) for 1 h and then co-exposed to LPS (1 µg/ml) for further 24 h for detection of nitrite (NO_2_^−^), iNOS and COX-2 expression and for 10 minutes for detection IkB-α expression (in mouse peritoneal macrophages).

NO generation was measured as NO_2_^−^, index of NO released by cells, in the culture medium 24 h after LPS stimulation by Griess reaction, as previously reported [26]. Briefly, 100 µL of cell culture medium were mixed with 100 µL of Griess reagent – equal volumes of 1% (w∶v) sulphanilamide in 5% (v∶v) phosphoric acid and 0.1% (w∶v) naphtylethylenediamine-hydrogen chloride (HCl) and incubated at room temperature for 10 min, and then the absorbance was measured at 550 nm in a microplate reader Titertek (Dasit, Cornaredo, Milan, Italy). The amount of NO_2_^−^, as µM concentration, in the samples was calculated by a sodium NO_2_^−^ standard curve. iNOS and COX-2 protein expression in J774A.1 and IkB-α expression in mouse peritoneal macrophages was assessed by Western blot analysis. After NO_2_^−^ determination cells were scraped off, washed with ice-cold phosphate-buffered saline (PBS), and centrifuged at 5.000 g for 10 min at 4°C. The cell pellet was lysed in a buffer containing 20 mM Tris HCl (pH 7.5), 1 mM sodium orthovanadate, 1 mM phenylmethylsulfonyl fluoride, 10 µg/ml leupeptin, 10 mM sodium fluoride, 150 mM sodium chloride, 10 mg/ml trypsin inhibitor, and 1% Tween-20. Protein concentration was estimated by the Bio-Rad protein assay using bovine serum albumin as standard. Equal amounts of protein (50 µg) were dissolved in Laemmli's sample buffer, boiled, and run on a SDS polyacrylamide gel electrophoresis (SDS-PAGE) minigel (8% polyacrylamide) and then transferred to hybond polyvinylidene difluoride membrane for 40 min. at 5 mA cm2 into 0.45 µm. Membranes were blocked for 40 min in PBS and 5% (w/v) nonfat milk and subsequently probed overnight at 4°C with mouse monoclonal anti-iNOS, anti-COX-2 antibody (BD Laboratories), anti IkB-α or anti-tubulin (Santa Cruz Biotechnologies) in PBS, 5% w/v non fat milk, and 0.1% Tween-20. Blots were then incubated with horseradish peroxidase conjugated goat antimouse immunoglobulin (Ig)G (1∶5.000) for 1 h at room temperature. Immunoreactive bands were visualized using electro-chemiluminescence assay (ECL) detection system according to the manufacturer's instructions and exposed to Kodak X-Omat film. The protein bands of iNOS, COX-2, IkB-α and tubulin on XOmat films were quantified by scanning densitometry (Imaging Densitometer GS-700 BIO-RAD U.S.A.). Data are normalized with tubulin expression, used as reference protein, and expressed as arbitrary densitometric units as previously reported [27].

### TNF-α and IL-6 determination

TNF-α and IL-6 concentrations in J774A.1 and mouse peritoneal macrophages culture medium stimulated for 18 h with LPS (1 µg/ml) and IS (1000–62.5 µM) as previously described were assessed by an Enzyme-Linked Immuno Sorbent Assay (ELISA) assay by using a commercial kit, for murine TNF-α or IL-6, according to manufacturer's instruction (e-Biosciences, CA, USA). Results are expressed as pg/ml and ng/ml for TNF-α and IL-6 respectively.

### Immunofluorescence analysis with confocal microscopy

For immunofluorescence assay, J774A.1 cells (1×10^6^ per well) were seeded on coverslips in 12 well plate and treated with IS using two medium IS uremic concentrations (500–250 µM) for 1 h and then simultaneously with LPS (1 µg/ml) for 20 min. Then cells were fixed with 4% paraformaldehyde in PBS for 15 min and permeabilized with 0.1% saponin in PBS for 15 min. After blocking with BSA and PBS for 1 h, cells were incubated with Rabbit anti-phospho p65 antibody (Santa Cruz Biotechnologies) for 2 h at room temperature. The slides were then washed with PBS for three times and fluorescein-conjugated secondary antibody (FITC) was added for 1 h, DAPI was used for counterstaining of nuclei. Coverslips were finally mounted in mounting medium and fluorescent images were taken under the Laser Confocal Microscope (Leica TCS SP5).

### Measurement of intracellular Ca^2+^signalling

Intracellular Ca^2+^concentrations [Ca^2+^]i were measured using the fluorescent indicator dye Fura 2-AM, the membrane-permeant acetoxymethyl ester form of Fura 2, as previously described [28] with minor revisions. Briefly, J774A.1 cells (5×10^5^/multiwell 24 culture dishes) were incubated at 37°C. Thereafter, the medium was replaced with fresh medium and IS (1000–62.5 µM) was added. After incubation period (30 minutes or 2 hours), cells were washed in phosphate buffered saline (PBS), re-suspended in 1 ml of Hank's balanced salt solution (HBSS) containing 5 µM Fura 2-AM for 45 min. Thereafter, cells were washed with the same buffer to remove excess Fura 2-AM and incubated in Ca^2+^-free HBSS/0.5 mM EGTA buffer for 15 min to allow hydrolysis of Fura 2-AM into its active-dye form, Fura 2. J774A.1 cells then were transferred to the spectrofluorimeter (Perkin-Elmer LS-55). In another set of experiments, cells were pre-treated with IS for 1 h and then co-treated with LPS and IS (1000–62.5 µM) for 15 minutes. Treatment with ionomycin (1 µM final concentration), thapsigargin (Tg, 1 µM final concentration) or with carbonyl cyanide p-trifluoromethoxy-pyhenylhydrazone (FCCP, 0.05 µM final concentration) was carried out by adding the appropriate concentrations of each substance into the cuvette in Ca^2+^-free HBSS/0.5 mM EGTA buffer. In the experiments involving the use of Tg, the irreversible and selective inhibitor of the sarco(endo)plasmic reticulum Ca^2+^ATPase (SERCA), or of FCCP, a mitochondrial uncoupler used for mitochondrial calcium depletion, these compounds were added to the medium for 10 or 5 min, for Tg and FCCP respectively, before the beginning of the recordings, as previously described [28]. The excitation wavelength was alternated between 340 and 380 nm, and emission fluorescence was recorded at 515 nm. The ratio of fluorescence intensity of 340/380 nm (F340/F380) was used to estimate intracellular free calcium. Results are indicated as delta increase of fluorescence ratio (F340/F380 nm) induced by ionomycin - basal fluorescence ratio (F340/F380 nm).

### iNOS and COX-2 detection in peritoneal macrophages

Mouse peritoneal macrophages were plated into 6-well plates (5.0×10^4^ cells/well). Peritoneal macrophages were allowed to grow for 24 h, thereafter the medium was replaced with fresh medium and cells were treated with IS (500–250 µM) for 1 h and then exposed simultaneously to IS and LPS (1 µg/ml) for further 24 h. Macrophages were then collected, washed twice PBS and then incubated in Fixing Solution for 20 minutes at 4°C and then incubated in Fix Perm Solution for 30 minutes at 4°C. Anti-iNOS and anti-COX-2 were then added for further 30 minutes. The secondary antibody were added in Fix solution and cells fluorescence was evaluated using a fluorescence-activated cell sorting (FACSscan; Becton Dickinson) and elaborated with Cell Quest software.

### Data analysis

Data are reported as mean±standard error mean (s.e.m.) values of at least three independent experiments. Statistical analysis was performed by analysis of variance test, and multiple comparisons were made by Bonferroni's test. A *P*-value less than 0.05 was considered significant.

## Results

### IS doesn't affect macrophage viability

To elucidate the influence of IS on viability of J774A.1 and mouse peritoneal macrophages under our experimental conditions, cells were treated with IS (1000–62.5 µM) for 24 h. Our data indicated that viability of J774A.1 macrophages was not affected by IS treatment (data not shown).

*IS enhances ROS release from macrophages: effect of DPI and NAC*

To investigate whether IS mediates oxidative stress in J774A.1 macrophages, we evaluated its effect on intracellular ROS by flow cytometry. Kinetic studies showed that IS, at all tested concentrations (1000–62.5 µM), induced a significant ROS production in macrophages already after 10 min, reached its maximal effect after 1 h (data not shown).

**Figure 1:**
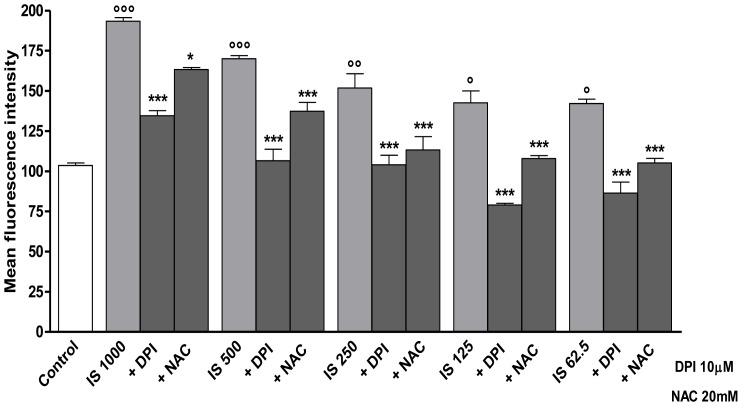
Indoxyl sulphate induces ROS formation in J774A.1 macrophages, effect of DPI and NAC. ROS formation was evaluated by means of the probe 2′,7′ dichlorofluorescein-diacetate (H2DCF-DA) in J774A.1 stimulate with IS (1000–62.5 µM) for 1 h. Where indicated, DPI (10 µM) or NAC (20 mM) for 30 min before to IS. ROS production was expressed as mean±s.e.m. of mean fluorescence intensity of six independent experiments. Data were analyzed by analysis of variance test, and multiple comparison were made by Bonferroni's test. *** denotes P<0.001 versus IS alone; °°°, °° and ° denote P<0.001, P<0.01 and P<0.05 respectively versus control.

After 1 h of incubation IS significantly increased ROS production in macrophages by 91.0%,65.6%, 47.9%, 38.9% and 38.5% (P<0.001 vs. control) at 1000, 500, 250, 125 and 62.5 µM, respectively (Fig. 1).

To study the mechanisms of IS-induced ROS in J774A.1 macrophages, we examined the potential role of the pro-oxidative enzymes NAD(P)H oxidase. ROS production in macrophages was measured after treatment with IS (1000–62.5 µM), in the presence of DPI, a NAD(P)H oxidase inhibitor. DPI significantly inhibited ROS release induced by all concentrations of IS (P<0.001 vs IS alone; Fig. 1). We also analyzed whether the addition of the antioxidants NAC could decrease oxidative stress caused by IS. As shown in Fig. 1, the IS induced ROS production was strongly inhibited by NAC (P<0.05 vs IS alone), although to a lesser extent when compared to DPI inhibition.

### IS modifies intracellular regulation of Ca^2+^ homeostasis, mainly influencing the mitochondrial compartment

**Figure 2:**
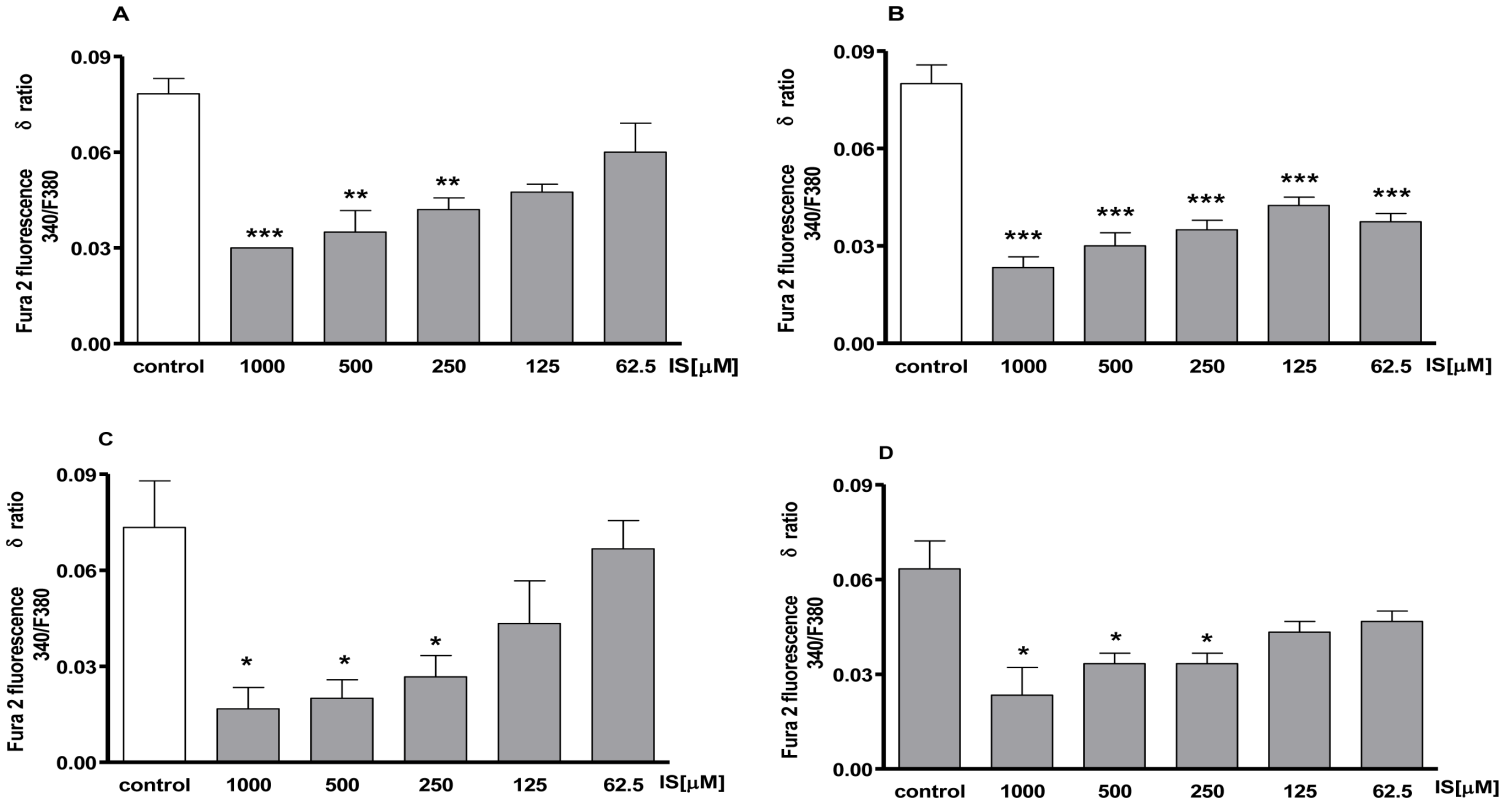
Concentration – related effect of IS on [Ca^2+^]i concentrations. Intracellular calcium concentration was evaluated on J774A.1 cells in Ca^2+-^free medium 30 minutes (panel A) or 2 h (panel B) after IS treatment (1000–62.5 µM). Effect of IS on mitochondrial Ca^2+^ pool was evaluated on J774A.1 cells in Ca^2+^-free medium in presence of FCCP (0.05 µM) added for 30 minutes (panel C) or 2 h (panel D) after IS treatment. Results were expressed as mean±s.e.m. of delta (δ) increase of FURA 2 ratio fluorescence (340/380 nm) from three independent experiments. Data were analyzed by ANOVA, and multiple comparison were made by Bonferroni's test. ***, ** and * denote P<0.001, P<0.01 and P<0.05 respectively versus control cells (J774A.1 in medium without LPS nor IS).

To avoid contribution of intracellular Ca^2+^ to IS-evoked signals we carried out experiments in Ca^2+^-free incubation medium (containing 0.5 mM EGTA). In the Ca^2+^-free medium, IS (1000-62.5 µM)-treatment significantly increased basal level of [Ca^2+^]_i_ -in a dose- and time-dependent manner. In fact, ionomycin administration evoked a small increase in [Ca^2+^]_i_ in IS treated cells both after 30 min and after 2 h of incubation (P<0.001; Fig. 2A, 2B).

To investigate the source of Ca^2+^release under the influence IS, J774.A1 cells were incubated for 5 min with FCCP (0.5 µmol/L), a mitochondrial calcium depletor, or for 10 min in the presence of Tg (1 µmol/L) which depletes the intracellular calcium stores, in a Ca^2+^-free medium. To analyze the involvement of mitochondrial Ca^2+^content in IS-evoked responses, cells were incubated with IS (1000–62.5 µM) and then for 5 min with 0.5 µmol/L FCCP, in the absence of extracellular Ca^2+^ to deplete mitochondrial Ca^2+^ content and then ionomycin was added. Figure 2C, 2D show that in the presence of FCCP delta increase in [Ca^2+^]_i_ was significantly (P<0.05) reduced in J774.A1 cells treated with IS, both for 30 min and for 2 h, in a dose- and time-dependent manner. In the presence of Tg, no significant differences in delta increase in [Ca^2+^]_i_ among IS- treated and control cells were observed (data not shown).

### IS enhances LPS-induced ROS release and increases mitochondrial superoxide production

To verify the effect of IS on ROS release in LPS-stimulated macrophages we evaluated intracellular ROS by incubating J774A.1 with IS (1000–62.5 µM) 1 h before and simultaneously with LPS. After 24 h LPS induced a significant increase of ROS release (2.06±0.10 fold increase vs control; P<0.01). IS at the higher concentration tested, in presence of LPS (1 µg/ml), significantly and in a concentration-related manner further increased ROS release respect to macrophage treated with LPS alone (2.1±0.11 fold increase for 1000 µM, P<0.01 vs LPS alone, and 1.36±0.08 for 500 µM, P<0.05 vs LPS alone).

**Figure 3:**
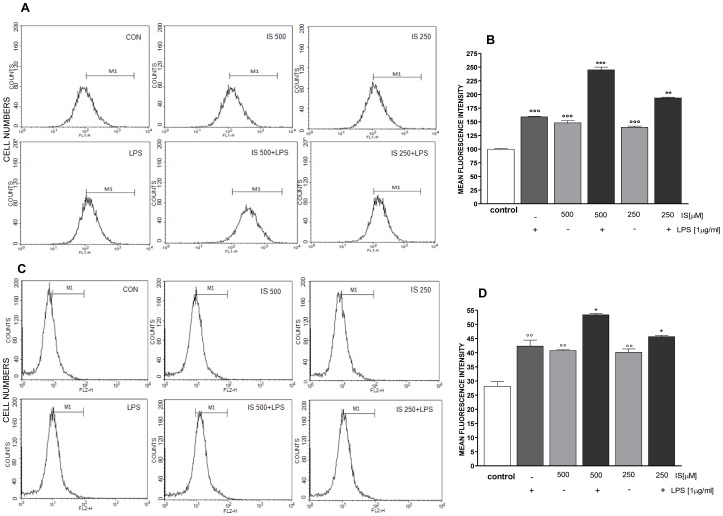
IS increases ROS production in LPS treated J774A.1 macrophages. ROS formation was evaluated by means of the probe 2′,7′ dichlorofluorescein-diacetate (H2DCF-DA) in J774A.1 cells. IS (500–250 µM) was added 1 h before and simultaneously to LPS (1 µg/ml) for 15 minutes. Panel A reports representative histograms for ROS detection. In panel B, ROS production was expressed as mean±s.e.m. of mean fluorescence intensity six independent experiments. In the same experimental conditions we also evaluated superoxide production by mitochondria adding MitoSOX Red. Panel C reports representative histograms for mitochondria superoxide detection. In panel D, mitochondrial superoxide production was expressed as mean±s.e.m. of mean fluorescence intensity of at least three independent experiments. Data were analyzed by analysis of variance test, and multiple comparison were made by Bonferroni's test. *** and ** denote P<0.001 and P<0.01 respectively versus LPS; °°°denotes P<0.001 versus control.

ROS release from J774A.1 treated with IS (1000–62.5 µM) 1 h before and simultaneously with LPS resulted significantly increased already after 15 minutes from LPS (data not shown for all concentrations). In particular IS at 500–250 µM (the concentration range most frequent in CKD patients) significantly increased LPS-induced ROS from macrophages (P<0.01 vs LPS alone; Fig. 3A, 3B). Under the same experimental conditions we measured mitochondrial superoxides by means of flow cytometry. As shown in Figure 3C, 3D, after 15 min. LPS induced superoxide release from mitochondria resulted significantly increased by IS 500–250 µM (P<.0.05 vs LPS alone).

### IS rapidly enhances LPS- induced Ca^2+^ release from mitochondrial store

**Figure 4:**
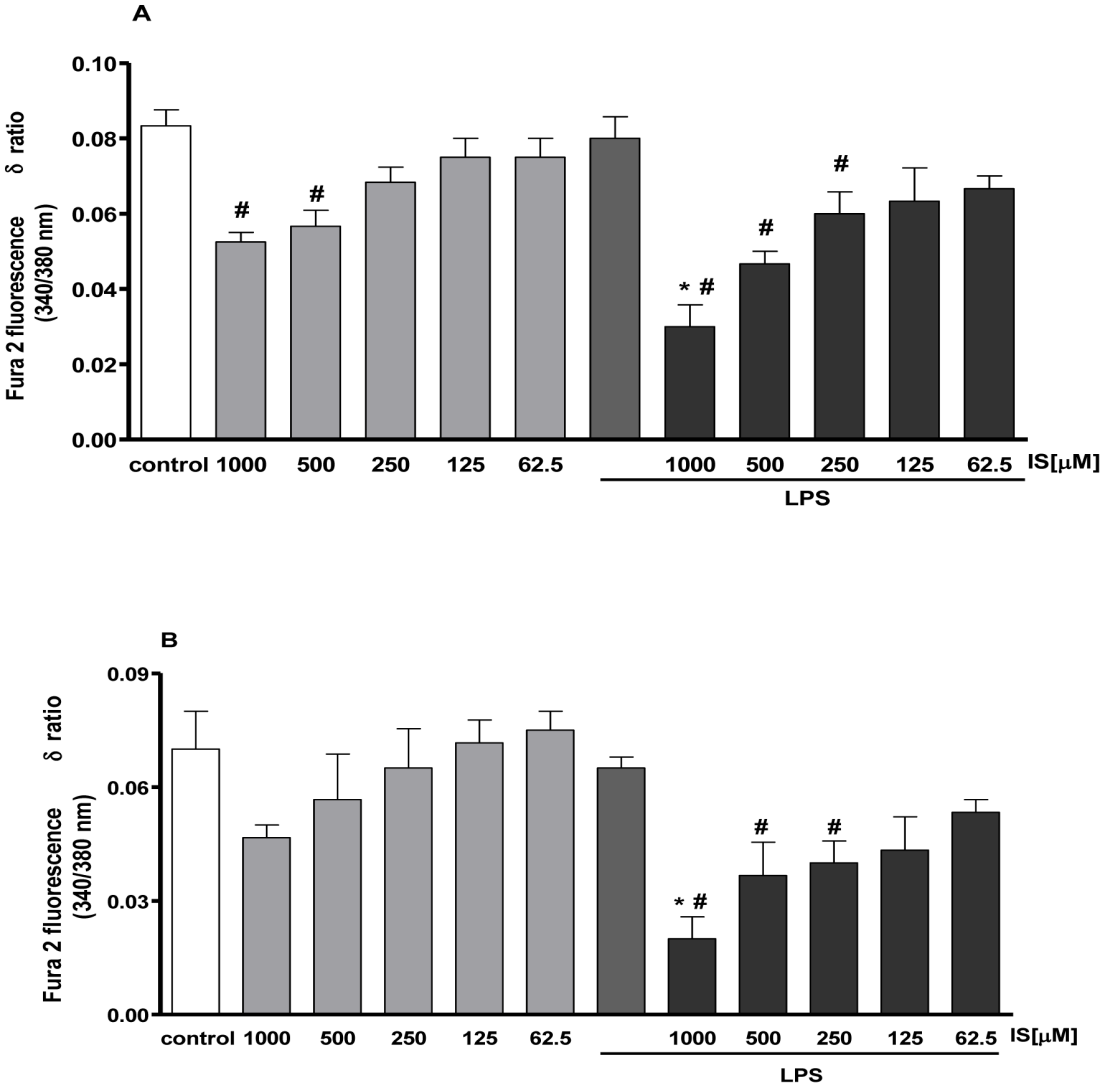
IS increase [Ca^2+^]i concentrations in LPS treated J774A.1 macrophages. Cells were pre-treated with IS for 1 h and then co-treated with LPS and IS (1000–62.5 µM) for 15 minutes. Intracellular calcium concentration was evaluated on J774A.1 cells in Ca^2+^-free medium (panel A). Effect of IS on mitochondrial Ca^2+^ pool was evaluated on J774A.1 cells in Ca2+-free medium in presence of FCCP (0.05 µM) (panel B) after LPS and IS treatment. Results were expressed as mean±s.e.m. of delta (δ) increase of FURA 2 ratio fluorescence (340/380 nm) from three independent experiments. Data were analyzed by analysis of variance test, and multiple comparison were made by Bonferroni's test. # denotes P<0.05 versus control cells (J774A.1 in medium without LPS or IS).

Pr-treatment with LPS (1 µg/ml) significantly enhanced calcium impairment. In fact, as shown in Figure 4, in LPS-treated cells [Ca^2+^]_i_ impairment was more evident even at lower concentration of IS. Furthermore, mitochondrial Ca^2+^ impairment was statistically significant (P<0.05 versus control) only in LPS and IS co-treated cells (Fig. 4B).

### IS facilitates p65 nuclear translocation induced by LPS

**Figure 5:**
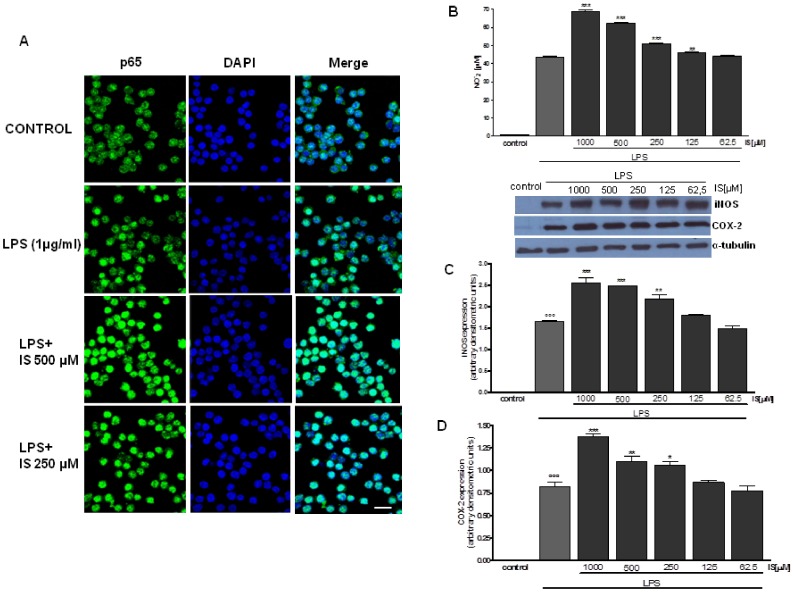
Effect of IS on LPS-induced p65 nuclear translocation, NO release and iNOS expression. J774A.1 cells were treated with IS (1000–62.5 µM) for 1 h and then co-exposed to LPS (1 µg/ml) for 20 min and nuclear translocation of NF-kB p65 subunit was detected using immunofluorescence assay at confocal microscopy. Scale bar, 10 µm. A representative of three experiments was shown (panel A). NF-kB activation is involved in NO release and iNOS protein expression. J774A.1 cells were treated with IS (1000–62.5 µM) for 1 h and then co-exposed to LPS (1 µg/ml) for further 24 h and NO (panel B) and iNOS (panel C) expression was detected by Griess reaction and Western blot respectively. Tubulin protein expression was used as loading control. Results are expressed as mean±s.e.m. from three independent experiments. Data were analyzed by ANOVA test, and multiple comparison were made by Bonferroni's test. °°° denotes P<0.001 versus control. ***, ** and * denote P<0.001, P<0.01 and P<0.05 resspectively versus LPS. Bar = 5 µM.

After p65 phosphorylation, the free NF-κB dimers translocate into the nucleus and bind to specific sequences to regulate downstream genes expression [29]. Thus we labelled p65 with green fluorescence to track the influence of IS (500-250 µM), added 1 h before LPS (1 µg/ml) on NF-κB translocation. As shown in Fig. 5a, nuclear p65 was increased after 15 minutes by LPS. NF-κB translocation is further enhanced by IS in J774A.1 treated macrophages when compared to LPS alone. IS alone had no effect on p65 nuclear translocation (data not shown).

### IS enhances NO release, iNOS and COX-2 expression from LPS-stimulated macrophage

To asses if IS influences NO production we measured NO_2_^−^ release, a stable end-product of NO, in cellular medium of the murine macrophage cell line J774A1 stimulated with IS (1000–62.5 µM) alone or in combination with LPS (1 µg/ml). A marked increase of NO_2_^−^ production in cellular medium was observed at 24 h after LPS (44.21±0.8 µM; P<0.001, vs control). When IS (1000–62.5 µM) was added to J774A.1 macrophages, 1 h before or together with LPS, a significant and concentration-dependent increase of NO_2_^−^ production in cell medium was observed by 68.9±0.60 µM P<0.001 vs LPS; 62.19±0.63 µM P<0.001 vs LPS; 51.16±0.5 µM P<0.001 vs LPS; 46.12±0.23 µM P<0.01 vs LPS respectively for 1000, 500, 250 and 125 µM. Only the lowest concentration tested (62.5 µM) didn't significantly increase NO_2_^−^ release (43.87±0.40 µM P<0.01 vs LPS; Fig. 5B). Under the same experimental conditions we also observed a significant induction in iNOS and COX-2 expression in macrophage treated with LPS alone (P<0.001 vs control). When IS (1000–62.5 µM) was added to J774 macrophages, 1 h before and simultaneously with LPS, a significant and concentration-dependent increase in iNOS expression was observed at concentrations between 1000–125 µM (P<0.05 vs LPS alone; Fig. 5C). Under the same experimental conditions COX-2 was also resulted, although to a lesser extent compared to iNOS, at concentrations between 1000 and 250 µM (P<0.05 vs LPS alone; Fig. 5D). No increase in NO release and in iNOS and COX-2 expression was observed in J774A.1 macrophages treated with IS alone for 24 h (data not shown).

### IS enhances LPS-induced cytokines production in macrophages

**Figure 6:**
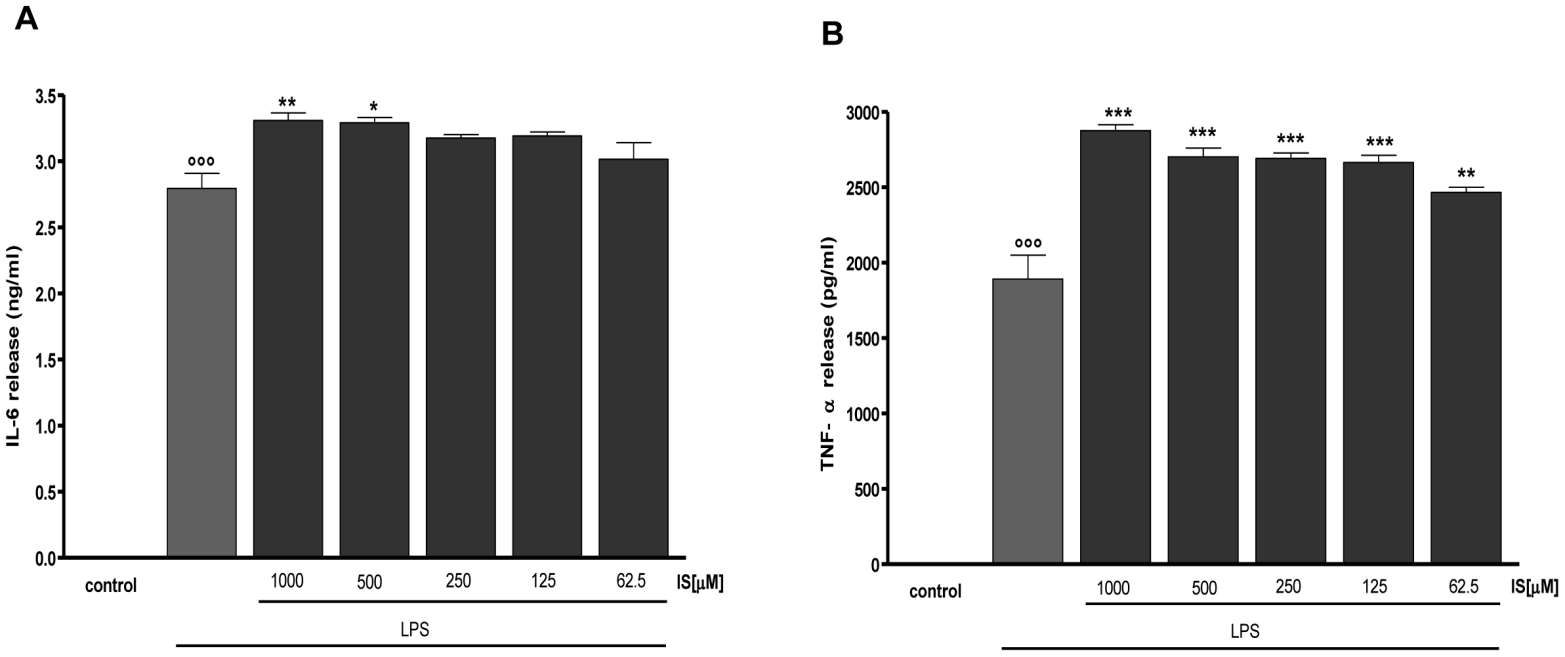
Effect of IS on LPS –induced IL-6 and TNF-α production in J774A.1 macrophages. IL-6 (panel A) and TNF-α (panel B) production was measured in the supernatants of J774A.1 cells treated with IS (1000–62.5 µM) and LPS (1 µg/ml) for 18 h by an ELISA kit. Results are expressed as mean±s.e.m. from three independent experiments. Data were analyzed by ANOVA test, and multiple comparison were made by Bonferroni's test. °°° denotes P<0.001 versus control. ***, ** and * denote P<0.001, P<0.01 and P<0.05 respectively versus LPS.

LPS induced a significant induction in TNF-α and IL-6 levels in J774A.1 macrophage (P<0.001 vs control; Fig. 6). This release was significantly enhanced by IS (1000–62.5 µM) added to cells 1 h before and simultaneously with LPS. In particular, in the case of IL-6 release we observed a significant increase induced by IS at the concentrations of 1000 and 500 µM (P<0.05 vs LPS alone; Fig. 6A). TNF-α release was significantly increased at all tested concentrations (P<001 vs LPS alone; Fig. 6B). IS alone didn't affect significantly cytokines release from macrophages (data not shown).

### IS enhances inflammatory response in LPS-stimulated mouse peritoneal macrophages

**Figure 7:**
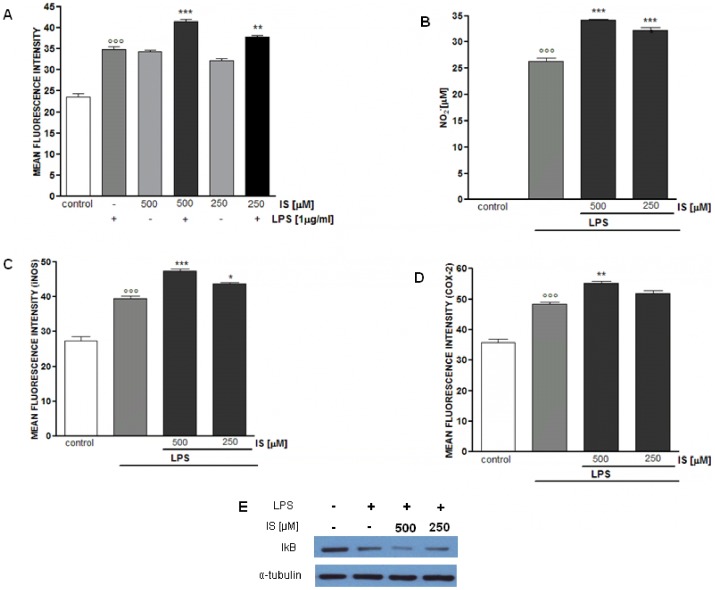
Effect of IS on murine peritoneal macrophages. ROS formation was evaluated by H_2_DCF-DA in peritoneal macrophages treated with IS (500–250 µM) for 1 h. Where indicated IS was also added together with LPS (1 µg/ml) for 15 minutes (panel A). Peritoneal macrophages were treated with IS (500–250 µM) for 1 h and then co-exposed to LPS (1 µg/ml) for further 24 h to assess NO release (panel B), iNOS (panel C) and COX 2 (panel D) expression. In order to evaluate the effect of IS on NF-kB pathway macrophage were treated with IS for 1 h, then together with LPS for 10 minutes and IkB- α degradation was assed by Western blot, a representative of three experiments was shown (panel E). Results are expressed as mean±s.e.m. from three independent experiments. Data were analyzed by ANOVA test, and multiple comparison were made by Bonferroni's test. °°° denotes P<0.001 versus control. ***, ** and * denote P<0.001, P<0.01 and P<0.05 respectively versus peritoneal macrophages treated with LPS alone.

To investigate if the observed effect of IS in J774A.1 macrophages were confirmed in primary macrophages we evaluated the effect of IS (500–250 µM) on ROS, NO, iNOS, COX-2 and IkB- α in murine peritoneal macrophages both in basal conditions and in the presence of LPS (1 µg/ml). IS alone after 1 h induces a significantly increase in ROS release from macrophages. Moreover IS also significantly increased LPS-induced ROS from macrophages in a concentration-dependent manner (P<0.01vs LPS alone; Fig.7A). When peritoneal macrophages were treated with IS for 1 h and together with LPS for further 24 h a significant and concentration-dependent increase in NO_2_^−^ production was observed (P<0.001 vs LPS alone; Fig.7B). Similarly also LPS-induced iNOS and COX-2 expression resulted increased by IS. In particular IS significantly increases iNOS expression at both concentrations tested (P<0.05 vs LPS alone; Fig. 7C) while affecting COX-2 expression only at the highest tested concentration (P<0.01 vs LPS; Fig. 7D). In peritoneal macrophages LPS induced a significant TNF-α release (48.3±2.3 ng/ml; P<0.001 vs untreated cells) which resulted further increased by IS treatment (75.6±3.4 ng/ml and 70.9±2.3 ng/ml respectively for 500 and 250 µM P<0.001 vs LPS alone). In its unstimulated form, NF-kB is present in the cytosol bound to the inhibitory protein IkB. After induction of cells by a variety of agents, such as LPS, IkB becomes phosphorylated and triggers a proteolytic degradation of IkB; then, NF-kB is released from IkB and translocated to the nucleus [29]. After 10 minutes LPS induced IkB degradation which resulted increased by IS in a concentration-dependent manner (Fig. 7E). No increase in all evaluated parameters was observed in murine peritoneal macrophages treated with IS alone for 24 h (data not shown).

## Discussion

Uremia-related immune dysfunction result from a complex interaction between the innate and adaptive immune systems, in which immune activation (hypercytokinemia and acute-phase response) and immune suppression (impairment of response to infections and poor development of adaptive immunity) coexist. On the one hand, as a consequence of tissue damage, the innate immune system is triggered, and although inflammation in principle is an essential response to eliminate aggressors, it can be considered a double-edged sword when the initial reaction is not limited [11]. Interestingly, the main causes of death in patients with CKD are related to infectious and cardiovascular diseases, both being pathologic processes closely linked to immune function [7]. Therefore, accelerated tissue degeneration (as a consequence of chronic inflammation) and increased rate of sepsis (because of a poorly orchestrated immune response) represent the most important targets for interventions aiming to reduce mortality in CKD patients. Factors related to uremic toxicity, independent from dialysis, as a trigger of immune response in CKD has been highlighted. Reduction of renal function *per se,* and consequently uremic toxicity, can be responsible for increased plasma concentrations of systemic and vascular inflammatory biomarkers [29]–[31]. Despite the development of new technologies of renal replacement therapy, it is almost impossible to completely remove the uremic toxins retained by impaired renal function. The uremic toxins consist of heterogeneous substances, including organic compounds and peptides, with proinflammatory effects [16]. As for other immune cells, macrophage function is also inhibited by uremic toxins [16]–[20]. IS is mainly known as a nephrovascular toxin and no studies, to our knowledge, have reported its effect during inflammatory conditions. The main findings of this study is that IS is a proinflammatory uremic toxin in LPS-stimulated macrophages. Our results provide evidence that, in J774A.1 macrophages, IS induces i) ROS production, ii) and calcium release, mostly from the mitochondrial compartment enhancing the inflammatory conditions. Moreover in the presence of LPS these effects are further increased. In particular IS treatment also led to an increase of LPS-induced iii) NF-kB activation, iii) NO release, iv) iNOS and COX-2 expression and v) pro-inflammatory cytokine (TNF-α and IL-6) release. Moreover ROS, NO and TNF-α levels, iNOS and COX-2 expression and IkB-α degradation resulted induced by IS also in primary LPS-stimulated peritoneal macrophages. All these findings support the view that IS contributes to the deleterious effect associated to inflammation in CKD patients.

Oxidative stress is a feature of CKD patients and is commonly considered associated with an excessive ROS production due to an imbalance between pro- and anti-oxidant cellular mechanisms [13]. In this study we report that IS induces a rapid and significant increase in ROS release from macrophage reflecting an induction of oxidative stress state. One of the most immediate response of monocytes/macrophages to a variety of activating stimuli is the production of potent oxygen derived radicals, as superoxide anion mainly produced by NAD(P)H oxidase complex. Our experiments show that IS-induced ROS could be decreased in the presence of the NAD(P)H inhibitor DPI. In addition also the presence of NAC, an antioxidant which is a precursor of reduced glutathione [32], was able to reduce ROS induction by IS in macrophages. In support to our data, previous studies reported that IS induces oxidative stress by interfering both with pro- and anti-oxidant factors in endothelial cells [33], [34], vascular smooth muscle cells [35] and kidney cells [36]. Moreover it has been reported that NAD(P)H oxidase resulted increased in CKD patients and in experimental models of renal insufficiency [37], [38].

CKD is also associated to calcium homeostasis alteration [39] and its intracellular alterations are been implicated in the control of a large variety of processes, including superoxide production via NAD(P)H oxidase, NO production and NF-kB activation [40], [41]. Here we show that IS induces a significant increase in basal level of [Ca^2+^]_i_, in a dose- and time-dependent manner in macrophages. These results reflect what found in polymorphonuclear leukocytes from patients undergoing haemodialysis. PMNLs induced higher release of basal [Ca^2+^]_i_ compared to healthy cells [39].

Sepsis is an heterogeneous class of syndromes caused by a systemic inflammatory response to infection. The rate of mortality from sepsis in critically ill patients is increasing despite improvements in supportive care [42].

Sepsis results from the generalized activation of inflammatory cascades following invasion of the blood stream by bacteria, viruses or parasites, with the systemic release of various toxic products. These products include bacterial cell-wall components, such as endotoxin as LPS, membrane component of gram-negative bacteria, able to induce systemic shock and finally death [43], [44]. Considering that infections and sepsis are frequent in CKD patients we evaluated the effects of IS in macrophages in this experimental condition. To mimic, as possible *in vitro*, the uremic condition we incubated J774A.1 macrophages with IS, at uremic concentrations, 1h before and simultaneously with the pro-inflammatory agents LPS for further 24 h. It is known that LPS has multiple effects on macrophages, such as regulation of their cellular functions, activation of transcription factors, production of cytokines and other pro-inflammatory mediators [20]. In macrophages LPS, within several minutes, induce ROS generation, constituting a critical factor in host defence mechanism. Alternatively, ROS activation could acts as a significant and adverse participant in abnormal inflammatory disease. This pathway is in fact significantly increased in J774A.1 macrophage by IS not only after 24 h but already after 15 min from LPS treatment. Moreover ROS analysis from macrophages treated with MitoSOX Red, a specific indicator of superoxide in the mitochondria of live cells, showed that mitochondria derived superoxide contribute to IS- induced ROS increase in presence of LPS after 15 min. LPS is known to activate pro-inflammatory transcription factor NF-kB, which is also regulated by a number of second messengers, including calcium and ROS [45]. One of the primary physiological functions of NF-kB is the regulation of immune responses, including pro-inflammatory enzyme production (e.g. iNOS, COX-2), antigen presentation, pattern recognition and phagocytosis. Our data indicate that in presence of LPS IS enhances the phosphorylation and nuclear translocation of the p65 subunit thus enhancing NF-kB activity in J774A.1 macrophage. These results are in accordance with studies reporting the effect of IS in stimulating NF-kB pathway [21], [46] even though in our experimental condition we didn't observe NF-kB activation in macrophages treated with IS alone but only in co-presence of IS with LPS.

LPS enhances NO formation, following the induction of iNOS, that has been implicated in the pathogenesis of shock and inflammation [47]. In fact, during inflammation, NO is mainly produced by iNOS, which expression resulted influenced both by ROS and NF-kB activation. NO has been implicated in several aspect of septic shock [48], [49]. In fact, inhibition of either the expression and activity of iNOS protein exerts beneficial effects in animal models of shock [50]. Our evidences indicate that IS further increase NO release and iNOS expression during LPS-induced inflammation in macrophage thus contributing to the excessive NO release-mediated damage in sepsis. NF-kB response element are also involved in the expression of COX-2 during inflammation and the expression of this enzyme was also influenced by NO [51]. COX-2 is induced also by LPS and is the predominant COX at sites of inflammation. Even though to a lesser extent respect to iNOS, also COX-2 resulted increased by IS in LPS-stimulated macrophages, thus contributing to the high inflammatory response.

TNF-α and IL-6 are cytokines elevated in sepsis both in humans and animals and their production is tightly related both to NF-kB activation and ROS levels. Moreover these cytokines can induce iNOS expression and large amounts of NO production [52], [53]. Although cytokine production is necessary for protection against pathogens and promote tissue repair, excessive release or decreased clearance, or both, can lead to organ failure and premature death. In our experimental model IS, added 1 h before and simultaneously to LPS, significantly increased, even at the lower concentration tested, TNF-α and, to a lesser extent, IL-6 release. These results agree with human and animal studies indicating that inflammatory cytokines, as TNF-α and IL-6, are increased in CKD patients with corresponding increases of protein catabolism, malnutrition and atherosclerosis [54], [55]. Moreover the effect of IS in increasing inflammatory response to LPS were also observed in primary macrophages. Our results indicate that IS is an uremic toxin able to significantly affect macrophage innate immune response through a strong increase of pro-inflammatory mediators in response to LPS. Moreover although recent studies indicated t systemic inflammatory reaction and oxidative stress as major mechanisms of vascular disease in patients with CKD [56], [57], the pathophysiology of cardiovascular disease in CKD has not completely been understood. Macrophages and their products here evaluated have an important role both in immune response and in cardiovascular complication associated with CKD. Persistent inflammation is, *per se*, a risk factor for CKD progression but may also modulate the impact of other vascular and nutritional risk factors in the toxic uremic milieu [58]. Understanding the mechanisms underlying the immune dysfunction that is peculiar to CKD generates a perspective to improve outcome in this group of patients. Therefore in addition to therapies, as antioxidant, aimed to reduce oxidative stress another interesting way would be to decrease the levels of pro-oxidant and pro-inflammatory uremic solutes, as IS in CKD patients by a very low protein dietary intake [59], [60] or by oral adsorbent as AST-120 [61], [62].
